# Is there a “safe” suction pressure in the venous line of extracorporeal circulation system?

**DOI:** 10.1177/0267659120936453

**Published:** 2020-07-04

**Authors:** Yuri M Ganushchak, Erik PJ Körver, Jos G Maessen

**Affiliations:** 1 Department of Cardiothoracic Surgery, Maastricht University Medical Center, Maastricht, The Netherlands; 2 Cardiovascular Research Institute Maastricht (CARIM), Maastricht University Medical Center, Maastricht, The Netherlands

**Keywords:** extracorporeal circulation, centrifugal pumps, suction pressure, degassing, gaseous emboli, patient safety

## Abstract

Successes of extracorporeal life support increased the use of centrifugal pumps. However, reports of hemolysis call for caution in using these pumps, especially in neonatology and in pediatric intensive care. Cavitation can be a cause of blood damage. The aim of our study was to obtain information about the cavitation conditions and to provide the safest operating range of centrifugal pumps. A series of tests were undertaken to determine the points at which pump performance decreases 3% and gas bubbles start to appear downstream of the pump. Two pumps were tested; pump R with a closed impeller and pump S with a semiopen impeller. The performance tests demonstrated that pump S has an optimal region narrower than pump R and it is shifted to the higher flows. When the pump performance started to decrease, the inlet pressure varies but close to −150 mmHg in the test with low gas content and higher than −100 mmHg in the tests with increased gas content. The same trend was observed at the points of development of massive gas emboli. Importantly, small packages of bubbles downstream of the pump were registered at relatively high inlet pressures. The gaseous cavitation in centrifugal pumps is a phenomenon that appears with decreasing inlet pump pressures. There are a few ways to increase inlet pump pressures: (1) positioning the pump as low as possible in relation to the patient; (2) selecting appropriate sized venous cannulas and their careful positioning; and (3) controlling patient’s volume status.

## Introduction

Extracorporeal circulation as an interdisciplinary science requires not only a deep knowledge of physiology but also the understanding and usage of the laws of hydrodynamics. A comprehensive understanding of the physiology of the reciprocal relations in the patient’s heart–lung machine interaction is absolutely necessary for a perfusionist to effectively maintain homeostasis during cardiac surgery and extracorporeal life support (ECLS). The recent explosive expansion of areas where ECLS methods can be applied further increases the significance of specialized knowledge of the interaction of mechanical systems and the human body.

Successes of ECLS of patients during the 2009-2010 H1N1 epidemic led to the expansion of ECLS applications.^1–5^ As a result, there have been significant advances in extracorporeal circulation technology. All parts of the extracorporeal circle: cannulas, pump types, and oxygenators have been improved. In addition, it increased the use of magnetic-driven volute centrifugal pumps for ECLS through a wide range of age groups of patients. However, repeated reports of hemolysis, especially in younger patients, call for caution in using these pumps in neonatology and in pediatric intensive care.^6–11^

Distinct to industrial pumps, which are designed to work at a single pump speed, pumps for an extracorporeal circulation are required to cover a wide range of flow rates. Furthermore, most of these pumps, especially axial and mixed-flow impeller pumps, are high-flow low-pressure pumps.^12,13^ Therefore, often in clinical practice, an extraordinary pump speed is necessary to perfuse against a high hydrodynamic resistance of the circuit. This increases shear stress in the pump.^14^ High shear stress can cause hemolysis^15^ and destruction of high molecular weight multimers like von Willebrand factor.^16,17^ It also stimulates platelet and fibrinogen to form thrombus.^16^

Another cause of blood damage is cavitation. Cavitation bubbles will collapse violently causing serious damage to blood cells.^18^ One of the causes of cavitation is using pumps with low suction pressure. Each industrial centrifugal pump has a parameter that describes the lowest pressure in the eye of the impeller above vapor pressure. This value is specific for the pump and described by the term Net Positive Suction Head required (NPSHr). The "net" in the term means "over and above" the suction head required to maintain the fluid as a liquid.^19^ It is presented as an absolute pressure and converted to the length units of the pump head. The suction pressure at the inlet of the pump (presented as NPSH available) can be used as an indirect indicator of cavitation. A 3% drop in the pump head with a gradual decrease of NPSH is accepted as evidence that cavitation is present and is considered the standard method determining NPSHr.^13,20^

The aim of the present study was to perform the NPSH analysis of two centrifugal pumps accepted in clinical practice to obtain information about the cavitation conditions and to provide the safest operating range of the chosen pumps.

## Materials and methods

**Figure 1.**
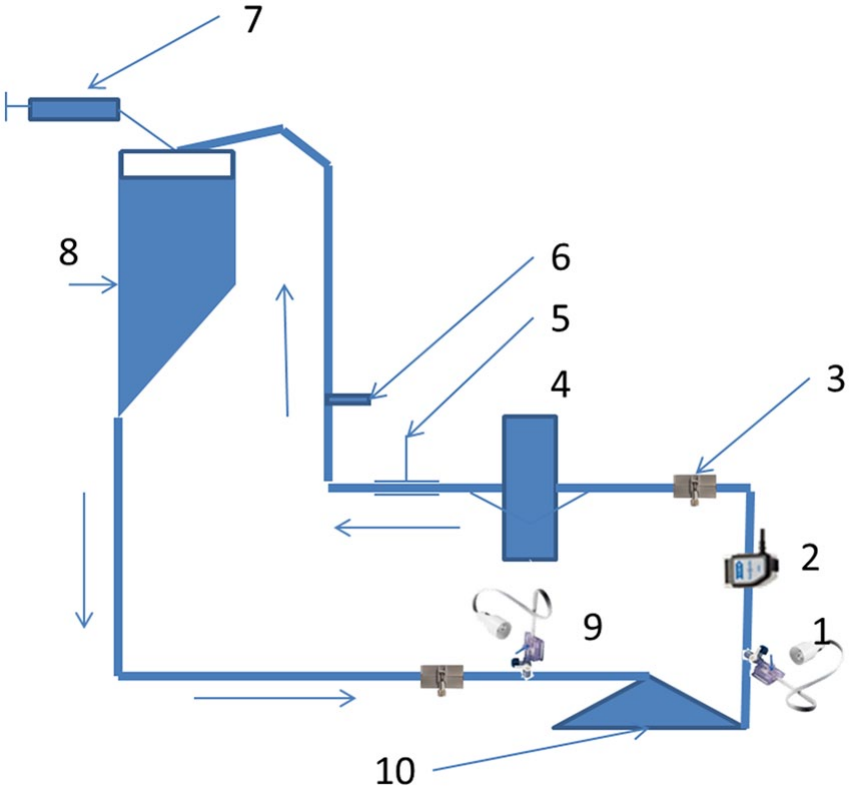
The schematic drawing of the test ring. 1—postpump pressure transducer; 2—flow sensor (H9XL, Transonic Systems Inc., Ithaca, NY, USA); 3—ultrasonic bubble sensor for measurements with 3/8″ tubes (GAMPT mbH, Merseburg, Germany); 4—hollow fiber oxygenator (The Quadrox-D-Adult oxygenator (Maquet Cardiovascular, Hirrlingen, Germany); 5—Hoffman clamp; 6—temperature probe; 7—vacuum source; 8—hard-shell reservoir; 9—prepump pressure transducer (TruWave™ disposable pressure transducers Edwards Lifesciences Corp., Irvine, CA, USA); 10—centrifugal pump.

A series of tests were undertaken to determine the NPSH3% of two centrifugal pumps, pump R with a closed impeller (50 mm diameter) and pump S with a semiopen impeller with a diameter of 60 mm. A test bench was designed at Maastricht UMC + for NPSH analysis of centrifugal pumps (Figure 1) as recommended by the Hydraulic Institute (http://www.pumps.org/).

Before the experiment, the system was primed to free it from bubbles and dissolved gasses. During the priming, fluid (water) was circulating at 4 L/min for 12 hours. The temperature was kept at 37°C, and the system was exposed to the subatmospheric pressure of −300 mmHg via the hydrophobic polymethylpentene hollow-ﬁber diffusion membrane of an oxygenator (Figures 1 and 4).

The pump performance test was conducted before the NPSH trials. The measurement of the NPSH3% value was made by reducing the system pressure by stepwise extracting air from the hard-shell reservoir at the flows that corresponded to the best efficiency point for the tested pump speed. By using this test procedure, the pump was kept at a constant flow rate and speed with the suction condition varied to produce cavitation.

There were two series of tests. In the course of the first test series, the water, as a test medium, was degassed during 12-hour recirculation under a subatmospheric pressure (only pump with closed impeller was used). The second set of measurements was done with water saturated by oxygen (2 L/min) given via the diffusion membrane of an oxygenator (both pumps were tested).

The tests were repeated twice for pump speeds of 1,000, 2,000, 3,000, 4,000, and 5,000 r/min for the pump R and 1,000, 1,500, 2,000, and 2,500 r/min for the pump S at the flows close to the flow at the best efficiency points (BEP) for each pump speed. The pump with semiopen impeller was not tested for pump speeds above 2,500 r/min because the flows at the estimated BEP at higher pump speeds were beyond measurable levels.

The bubble counter (BCC200, GAMPT mbH, Merseburg, Germany) with probes positionend before and after the pump was used for the detection of gas bubbles in the pumping fluid.

Flow and pressure were recorded by a data acquisition system with a sample rate of 250 Hz (M-PAQ, Instrument Development Engineering & Evaluation, Maastricht University Medical Centre, Maastricht, The Netherlands).

The total pump head as a representation of the work performed on the liquid by the pump and defined as

(1) $$TPH=\frac{P_{outlet}-P_{inlet}}{\rho *g}+\frac{{\left(Q/A\right)}^{2}}{2g}$$

where *P_outlet_* = pressure at the outlet of a pump (Pa); *P_inlet_* = pressure at the inlet of a pump (Pa); ρ = density (kg/m^3^); g = gravitational acceleration (m/s^2^); *A* = area of the tubing (m^2^); *Q* = volumetric velocity (m^3^/s).

The “pump head” is the basic characteristic of a pump, and in fact, this is the maximum height to which it can pump water used by manufacturers of industrial pumps because of the independency of parameters from the properties of the pumping liquid. The pump head in units of length can be converted to the pressure units (and vice versa) when the specific gravity of pumping fluid is known.

Net positive suction head (NPSH) is the suction head in the liquid pressure at pump suction above liquid vapor pressure. NPSH is a function of the system, and in engineering documentation, it is presented in terms of the height of the liquid column

(2) $$NPSH=\frac{p_{stat\left(in\right)}+p_{bar}-p_{v}+\left(0.5*\rho *v^{2}\right)}{\rho *g}$$

where *p_stat(in)_* = pressure at the suction side of a pump (Pa); *p_bar_* = barometric pressure (Pa); *p_v_* = liquid vapor pressure (Pa); ρ = density (kg/m^3^); v = velocity (m/s); g = gravitational acceleration (m/s^2^).

For industrial pumps, the minimum pressure required at the suction port of the pump to keep it from cavitating (NPSHr) measured as a 3% loss of total head due to cavitation at the BEP for this specific pump.

The BEP is the point along a pump curve where efficiency is highest. The overall efficiency of a centrifugal pump is simply the ratio of the hydraulic power to the input power. For practical purposes, the BEP can be defined as the duty point on the pump performance curves at 80-85% from the shut-off pump head.^21^ In an ideal scenario, a centrifugal pump should operate at or as close to the BEP as possible at all times (±10% from the BEP).

Another important parameter of centrifugal pump description is specific speed (Ns) which value is related to the impeller design.^13,22^ It specifies the way the liquid traverses and leaves the impeller blades. The commonly used equation for specific speed is as follows

(3) $$\mathrm{Ns}=\frac{N*\sqrt{Q}}{{\left(HDbep\right)}^{0.75}}$$

where Ns = specific speed ; *N* = pump shaft rotational speed (r/min); *Q* = flow rate at BEP (m^3^/s); *HD_bep_* = discharge head rise (m).

The computed specific speeds for the pumps under investigation were compared with standard values for the determination of the type of impellers.

The same pumping fluid with a constant temperature was used in all tests and the flow velocities were low, that is why it was possible to present results in the usual pressure units instead of meters of the pump heads.

## Results

**Figure 2.**
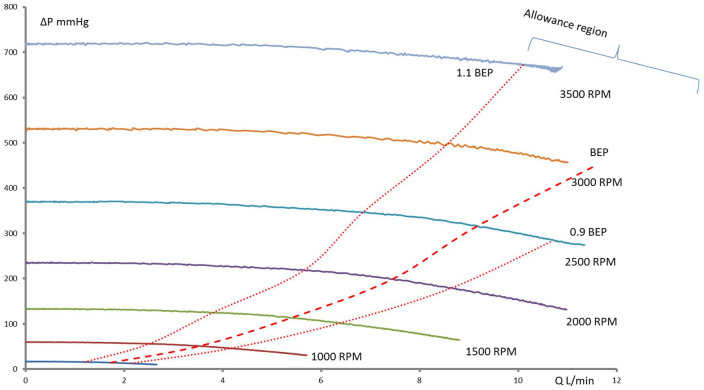
Pressure flow curves of pump S with semiopen impeller and a diameter of 60 mm. The dashed line connects BEPs at different pump speeds. The dotted lines show the borders of the allowance region.

**Figure 3.**
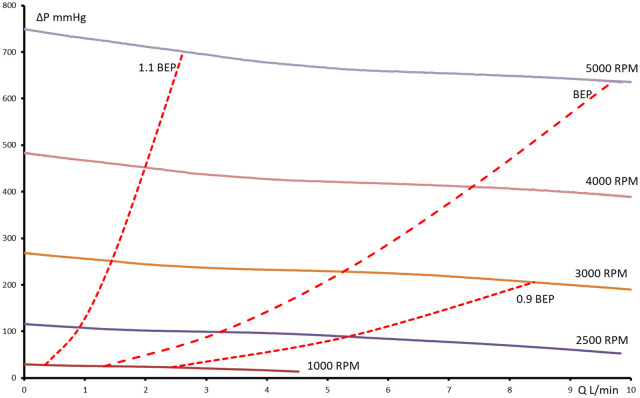
Pressure flow curves of pump R with closed impeller and a diameter of 50 mm. The dashed line connects BEPs at different pump speeds. The dotted lines show the borders of the allowance region.

The pump curves of the pump with the closed impeller and semiopen impeller are presented in Figures 3 and 2. The total pump head of pump curves is presented as pressure difference in mmHg between the inlet and outlet of the pump.

The pump with the semiopen impeller has a BEP shifting to higher flows in comparison to the BEP of the pump with the closed impeller. Also, the “allowance region” of the pump with the semiopen impeller is narrower. However, the specific speeds of both pumps in the study were similar and in the range of radial flow impellers (11.7 ± 0.8 and 10.1 ± 0.5, respectively, p > 0.05).

The BEP for the pump with the closed impeller was determined for all r/min (1,000-5,000 r/min, Figure 3) in the range of measurable flow (10 L/min). However, for the pump with the semiopen impeller, the BEPs were at flows higher than 10 L/min (Figure 2) at pump speeds of 3,000 and 3,500 r/min.

**Figure 4.**
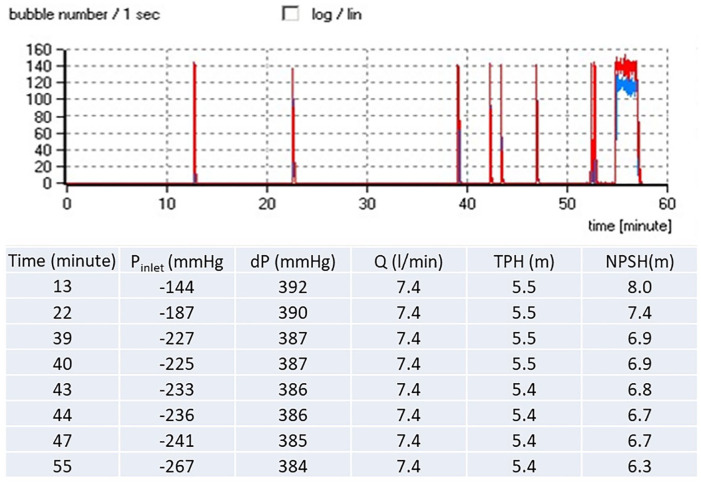
The example of small packages of gas bubbles in the flow, followed by massive gas production (pump R, 4,000 r/min, low gas content). Gaseous bubbles registered at the outlet of the pump are presented by red line. Gas bubbles registered at the inlet of the pump marked as blue line. *P_inlet_*—pressure at the inlet of pump; *dP*—pressure difference between inlet and outlet of the pump; TPH—total pump head; NPSH—net positive suction head.

Figure 4 shows an example of gas bubbles registration in the pumping fluid. The volume of bubbles as well as their number was much higher at the outlet of the pump than at the inlet.

**Figure 5.**
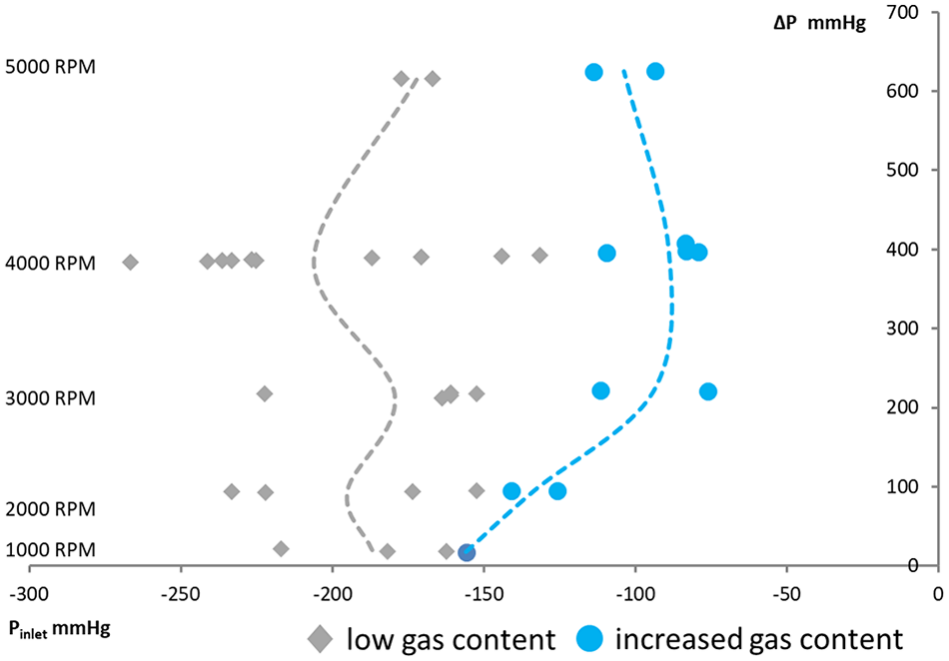
The pressure at the inlet of pump R and ΔP at the moment of gas bubbles registration. The gray rhombus marks correspond to the points of gas bubbles appearing in tests with low gas content and blue circles in tests with increased gas content. The dashed lines (gray for the tests with low gas content and blue for the test with increased gas content) are the points where the pump head was decreased at 3% from initial (pump R).

The same trend was seen in cases with increased gas content in the pumping fluid. However, gas bubbles appeared at the pressure higher than in cases with low gas content. The shift of gas bubbles “pressure” at the inlet of the pump as well as pressures representing NPSH3% to the right in the case of increased gas content in the pumping fluid is presented in Figure 5.

**Figure 6.**
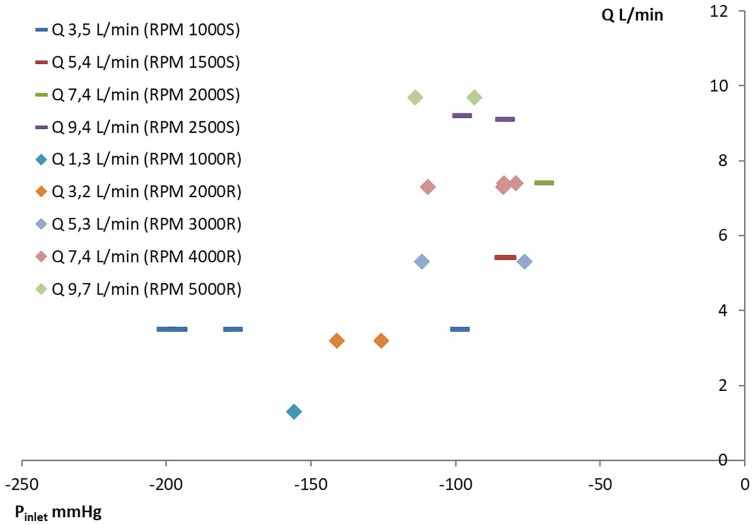
The pressure at the inlet of pumps at the moment of massive gas emboli (pump S and pump R with similar flows, tested with increased gas content).

Massive gas emboli in the case of pump R and pump S had the same flow and were registered at approximately the same pressures at the inlet of the pumps (Figure 6).

**Figure 7.**
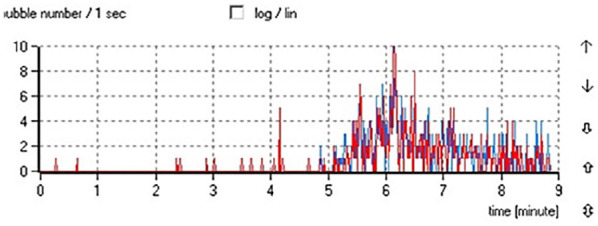
The example of small packages of gas bubbles downstream of the pump seen already from the inlet pressure of −10.6 mmHg and developed into the massive gas emboli at inlet pressure about −81 mmHg (≈4 min) (pump S, 1,500 r/min, Q 5.4 L/min, high gas content). Gaseous bubbles registered at the outlet of the pump are presented by red line. Gas bubbles registered at the inlet of the pump marked as blue line.

However, in tests in the pump with a semiopen impeller, small gas bubbles were registered occasionally already from the start of the test with relatively high pressures at the inlet of the pump (Figure 7).

## Discussion

The development of ECLS and minimized extracorporeal systems lead to not always recognized changes in the usage of centrifugal pumps. In standard extracorporeal circuits, the centrifugal pump takes blood from the reservoir where inflow is almost never restricted and inlet pressure is close to the atmospheric pressure. In minimized circuits, as well as in ECLS centrifugal pumps, blood is received directly from the venae via a cannula. Resistance of the cannula as well as sharp changes under the drainage condition causes the development of sub atmospheric pressures in the venous line and the centrifugal pump, development of gas bubbles,^23–25^ and direct damage of blood components. Correct exploitation of centrifugal pumps under these conditions requires measurement and control of pressure before the pump.^23,26–28^ However, the pressure in the venous line that can be recognized in clinical practice as safe is not clearly defined. In contrast, the allowable pressures at the inlet for industrial pumps in contrast to “medical” pumps are defined and are an obligatory part of their documentation in terms of NPSHr.^13,29^

The aim of our study was to find the critical NPSH for two centrifugal pumps with a different design of the impeller. The test bench was built with the theoretical principles from previously established studies on the performance of centrifugal pumps. For the experiment, the NPSH value was gradually decreased to study the start of cavitation and its development.^18,30–32^ This could be achieved by throttling the suction valve to reduce the total suction pressure, while properly regulating the discharge valve to retain the flow rate. However, this resulted in cavitation inception far before the pump’s inlet, in the suction valve, irrespective of the flow rate.^33^

Another way to decrease the suction pressure is a circuit with a closed tank in which the fluid level is held constant and the suction pressure is adjusted by varying the air or gas pressure over the liquid.^20^ This method was used in our study to avoid the effects of gas bubbles formed in the suction throttle. Another reason to use the close tank for regulating the pressure in the whole system was the expectation that lower outlet pressures will abate the process of gas bubbles implosion and will allow the registration of them downstream the pump.

Our results showed that the gas bubbles appeared downstream of the pump at pressures close to −150 mmHg in tests with decreased gas content and were shifted to values higher than 100 mmHg if the fluid was saturated by oxygen. The points of a 3% decrease in pump head demonstrated the same trend in the right-side shift with increased gas content. However, the pressures when the gas bubbles were registered downstream of the pump where variable and we could not find expected changes in critical inlet pressure with the pump speed (Figure 5). Interestingly, both pumps showed an episodic appearance of bubbles downstream the pump during a gradual decrease of pressure in the circuit and resulted in massive gas bubble production at lower pressures (Figures 4 and 7). We did not find any differences between the pumps R and S in inlet pressure resulting in massive gas bubble production (Figure 6). However, in tests with the semiopen impeller, pump episodic passing of small amounts of gas bubbles was seen almost from the start of the experiments (Figure 7).

The relatively high inlet pressure at the moment of gas bubbles registration downstream of the centrifugal pump as well as appearing of gas bubbles at the even higher inlet pressure with increasing gas volume dissolved in the pumping fluid could be explained only by the hydrodynamic cavitation. Besides the well-known formation of a vapor phase when the pressure in liquid falls below the effective vapor pressure, there exists a so-called gaseous cavitation. The gaseous cavitation is triggered when the pressure drops below the saturation pressure of the dissolved gas in the liquid (Henry’s law).^34^ Then, with a decrease of surrounding pressure and consequent growth of gas nucleus, the inside pressure of a gas nucleus decreases, making the dissolved gas around the gas nucleus to diffuse into a gas bubble, and further aggravating its growth.^35^ Despite the fact that diffusion of dissolved gas into the bubble during its inflation that is followed by dissolving and implosion of the bubble is a relatively slow process (in comparison to the vaporous cavitation), it generates high temperatures even with molecular-level cracking and degrades the chemical composition of the fluid through oxidation. The cavitation bubbles, generated in a low-pressure zone, will collapse violently, causing serious damage to blood cells.^18^

Another type of cavitation seen in pumps is due to a phenomenon called recirculation. Recirculation is defined as a flow reversal either at the inlet or at the outlet tips of the impeller vanes. This reversal causes a vortex that attaches itself to the pressure side of the vane. If there is enough energy available and the velocities are high enough, a damage will occur. There are two types that may occur together or separately: suction recirculation and discharge recirculation.^36^

The variability of our results in repeated tests could be related not only to the unintended changes in the gas content of pumping fluid but also to the appearance of recirculation with alteration of inlet and outlet pressures.

### Limitations of the study

The evaluation of pumps was done with water instead of blood as a pumping fluid. In clinical situations with blood, one may therefore expect a substantial aggravation of the described effects in our study. As blood is a special fluid that delivers oxygen to cells and transports blood carbon dioxide out, many gas nuclei are present, making the appearance of gaseous cavitation easier.^35^ Furthermore, the higher viscosity can result in the loss of pump efficiency^37,38^ and an increase in critical inlet pressure.^18^ Another factor specific to the blood is oxyhemoglobin, which releases oxygen into the plasma when its partial pressure decreases. It is clear that this continuous supply of gas into the plasma can further lead to an intensification of the process of gaseous cavitation in the pump. A decrease of venous line pressure triggers degassing of blood-dissolved gases and causes arterial gas microemboli that can become massive during persistent conditions of limited venous return.^28^

## Conclusion and recommendations

The process of gaseous cavitation in centrifugal pumps is a phenomenon that appears with decreasing inlet pressure. Our study suggests that there is no “safe” level of inlet pressure and it has to be kept as high as possible. This can be achieved by placing the pump as low as possible in relation to the patient, by selecting properly sized and carefully positioned venous cannulas, and by controlling the patient’s volume status.
